# Smog Sign: Hazy Diffusion-weighted Imaging Restriction in Dense Axonal Tracts in the Pons on Hyperacute MRI with Remarkable Clinical Improvement After Intra-arterial Thrombectomy

**DOI:** 10.7759/cureus.5461

**Published:** 2019-08-22

**Authors:** Asad Ikram, Mudassir Farooqui, Sajid Suriya, Syed A Quadri, Atif Zafar

**Affiliations:** 1 Neurology, University of New Mexico School of Medicine, Albuquerque, USA; 2 Neurology, Massachusetts General Hospital / Harvard Medical School, Boston, USA; 3 Cerebrovascular Medicine, University of New Mexico School of Medicine, Albuquerque, USA

**Keywords:** infarct, small vessel disease, basilar artery, locked-in syndrome, diffusion weighted imaging, magnetic resonance imaging

## Abstract

Patient selection is of prime significance when considering intra-arterial thrombectomy (IAT) for patients with basilar artery occlusion (BAO). We present a case of BAO with the clinical locked-in syndrome and “smoggy” appearance diffusion restriction on diffusion-weighted imaging (DWI) in the pons on a hyper-acute magnetic resonance imaging (MRI) scan.

A 62-year-old man was admitted with a National Institutes of Health Stroke Scale (NIHSS) of 21. An admission computed tomography angiogram (CTA) showed a mid-distal BAO. As the alteplase was being infused, the patient started declining with an examination suggesting locked-in syndrome. An emergent hyper-acute MRI showed true restricted diffusion on DWI at the entire right anterior inferior cerebellar artery territory, multiple punctate areas in the bilateral posterior inferior cerebellar artery territory, right > left hippocampus, and thalamic region mesial aspect, compatible with an acute posterior circulation infarct. He also had patchy or hazy pontine involvement with slight DWI signal changes but an impressive apparent diffusion coefficient (ADC) darkening in the bulk of pons. The patient was taken to the operating room (OR) for IAT within five hours of the witnessed stroke onset, successfully revascularized, and then discharged on the eighth day with NIHSS of 3. MRI at discharge showed a pronounced DWI restriction in the same areas involved in the hyperacute MRI done at admission. However, now the ADC hyper-intensity was less noticeable, with continued hazy and smoggy pontine signal changes on DWI. On the two-month follow-up, the patient had zero NIHSS and modified Rankin scale (mRS) scores, respectively.

DWI-MRI changes in acute ischemic stroke behave differently in tract areas with a density of nerve axons, manifesting as a hazy or smoggy appearance: the “smog sign” on DWI. In hyper-acute MRI, "hazy" or "smoggy" diffusion restriction on DWI in different axonal tract areas like the pons can correlate with good functional outcomes if successful reperfusion therapy is offered.

## Introduction

Basilar artery occlusion (BAO) is associated with high morbidity and mortality [1]. The failure of successful revascularization in acute BAO patients is a strong predictor of mortality [2]. Magnetic resonance imaging (MRI) is frequently utilized by centers for decision-making in patients presenting with BAO, as the bony structures of the skull base produce artifacts on Computed Tomography (CT) scans, thereby interfering with visualization of early ischemic changes [3-4]. Stroke neurologists and neuro-endovascular providers base their decision for intra-arterial thrombectomy (IAT) in BAO on various factors, ranging from the severity of clinical presentation, infarct load on MRI and the extent of pontine involvement [5-6]. However, if the MRI shows an evolving restriction in the pons with the clinical exam suggestive of locked-in syndrome or comatose state, the concern of a poor outcome can hinder the candidacy for IAT [5].

Recent trials on anterior circulation stroke have shown an improvement in outcome measures with IAT within six to 24 hours of stroke onset [7]. However, the treatment time window for the posterior circulation stroke is substantially longer than the anterior circulation stroke [8]. There is a scarcity of evidence-based data for the acute management protocols for patients with BAO. The challenge with BAO is multifold; from the varying and confusing clinical presentations making it a diagnostic challenge, to slowly evolving and fluctuating neurological exam changes in the ensuing hours. All these rationale and reasons result in delayed recanalization therapies (up to 24 hours) in BAO. Hence, the patient selection for IAT in BAO is of prime significance, with acute imaging playing a critical role in the decision-making, although the role of imaging for anterior circulation strokes is studied more than posterior circulation strokes [9]. In this case report, we present a patient with an acute BAO in a near locked-in state, showing “smoggy” diffusion-weighted imaging (DWI) restriction or brightening and apparent diffusion coefficient (ADC) darkening in the pons on a hyper-acute MRI scan. We propose that DWI-ADC changes on hyper-acute MRI in ischemic stroke behave differently in neuronal tracts having a density of nerve axons (corona radiata and pontine fibers), as they have a potential to regenerate.

## Case presentation

An otherwise healthy, 62-year-old man with no vascular risk factors was admitted for the witnessed onset of an acute change in the neurological exam. The patient was parking his truck on a farm when his friend noticed that he is slurring his words along with a noticeable right-sided weakness and inability to get off the truck without assistance. He was immediately taken to the nearest emergency room in a small rural hospital, where a stroke neurologist from a Comprehensive Stroke Center (CSC) provided a tele-stroke consultation. On the initial examination, the patient had a high National Institute of Health Stroke Scale (NIHSS) of 21 (somnolence (1), disorientation (2), task performance (1), partial gaze palsy overcome by effort (1), right hemiplegia (facial palsy-2, RUE-3, RLE-3), left-sided drift (LUE-1, LLE-1), mild sensory loss on right side (1), ataxia in bilateral limbs (2), dysarthria (2), and moderate aphasia (1)). His blood glucose was normal, and a non-contrast CT head was negative for an acute bleed. A stat CT angiogram (CTA) showed mid-distal basilar artery occlusion (BAO). Intravenous (IV) alteplase was initiated within three hours of the stroke onset. As alteplase was being infused, the patient’s mental status rapidly started to decline along with the respiratory compromise, a repeat head CT did not reveal any hemorrhage. His NIHSS worsened to 29 within 50 minutes of the alteplase infusion, and he became obtunded on examination, with unresponsive pupils, loss of consciousness (LOC), and low Glasgow Coma Scale (GCS) requiring intubation. He was emergently flown to the CSC, which took an hour.

**Figure 1:**
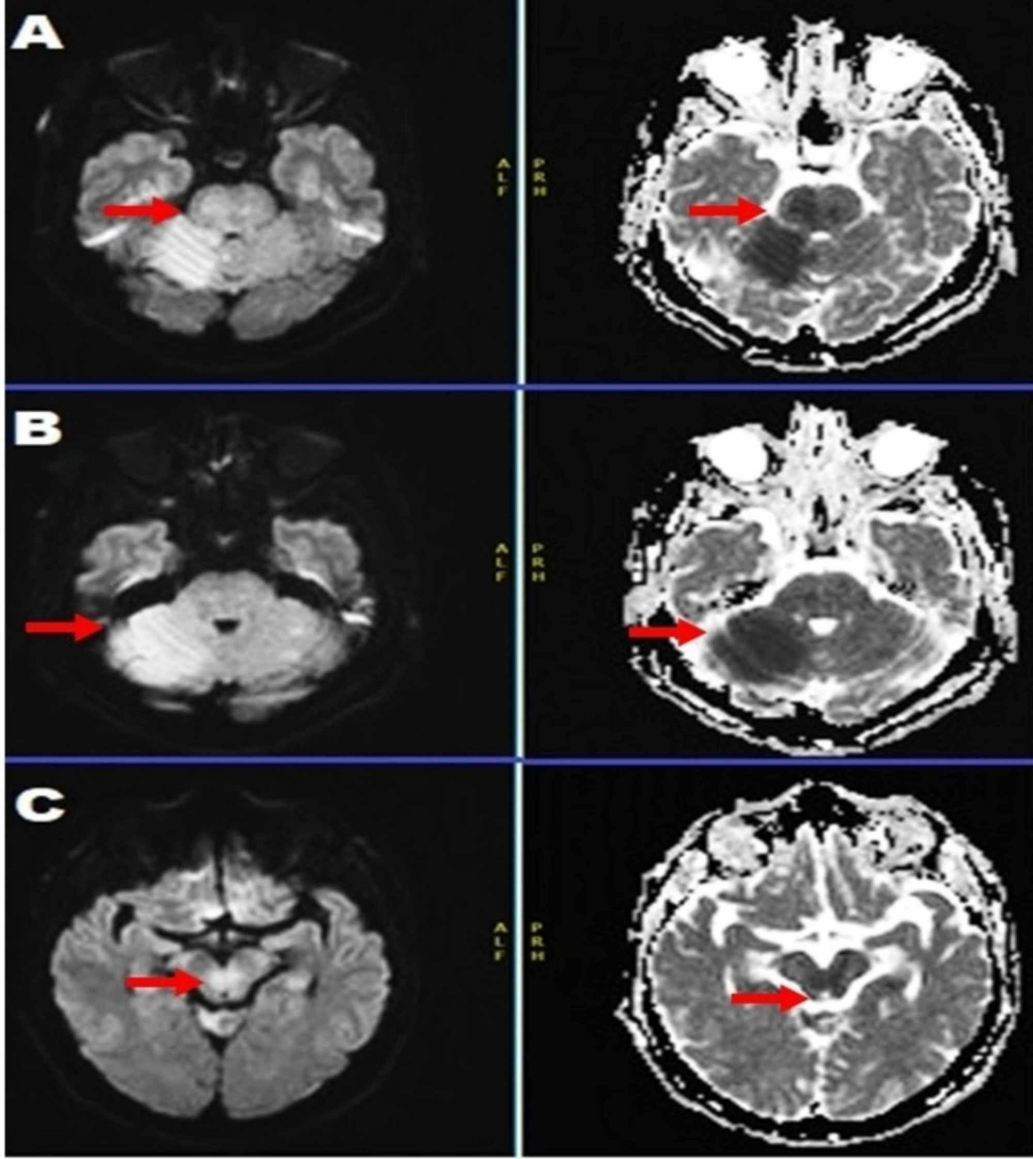
Multiple slices of MRI brain, diffusion-weighted images (DWI) obtained at five hours from awakening exhibited "smoggy" restricted diffusion with a noticeable apparent diffusion coefficient (ADC) correlation in the right more than left pons (red arrow) and cerebellar peduncle.

On arrival to the CSC, his NIHSS was 30 (somnolence (3), disorientation (2), task performance (2), partial gaze palsy overcome by oculocephalic reflex (1), right hemiplegia (facial palsy-2, RUE-4, RLE-3), no effort against gravity on left-side (LUE-3, LLE-3), complete coma (2), aphasia (3), and mute (2)); overall, the exam was suggestive of evolving and near-complete locked-in syndrome. The patient received an emergent hyper-acute MRI (four hours after the acute stroke onset), which showed true restricted diffusion on DWI at the entire right anterior inferior cerebellar artery (AICA) territory, multiple punctate areas at bilateral posterior inferior cerebellar artery (PICA) territory, right greater than left hippocampus and right more than left thalamic region mesial aspect, compatible with an acute posterior circulation infarct. He also had patchy or hazy pontine involvement with slight DWI signal changes but an impressive ADC darkening in the bulk of pons. With these findings, a concerning exam depictive of locked-in syndrome and family’s wishes to keep him on a Do Not Resuscitate/Do Not Intubate (DNR/DNI) status; there was some debate about not to intervene because the MRI lesions (ADC restriction in the pons, Figure 1) were predictive of a morbid outcome, which was against the patient’s wishes. After much discussion among the family and physicians (stroke, radiology, and endovascular teams), the patient was taken to the operating room (OR) for the IAT (within five hours of stroke onset), and successful revascularization of BAO was performed.

Details of the IAT procedure

The procedure went smoothly, with right femoral groin access, the subclavian injection demonstrated the normal course and caliber of the distal left vertebral artery, transient reflux of contrast down the right vertebral artery, as well as bilaterally patent anterior inferior cerebellar artery (AICA) and posterior cerebellar artery syndrome (PICA). BAO was seen just distal to the right AICA with an absent anterograde flow to the superior cerebellar arteries (SCA) and posterior cerebral arteries (PCA) bilaterally. Through manual suctioning, the basilar clot was successfully aspirated in the first pass within 30 minutes of the groin incision, with thrombolysis in cerebral infarction (TICI) 2b flow visualized on post-intervention angiogram. There was a small distal P4 segment occlusion on the left side.

Post-IAT, the patient stayed in a neuro-intensive care unit (ICU) for four days where a follow-up head CT scan did not show any hemorrhagic transformation. There were no major complications except some minor pseudobulbar behavioral changes that improved with the use of selective serotonin reuptake inhibitors (SSRIs). He was continued on aspirin and high-intensity statin. His neurological exam continued to improve with NIHSS of seven at the time of transfer to the step-down unit (SDU) for mild aphasia, disorientation, and motor right-sided weakness. Therefore, he was successfully extubated and transferred to the SDU.

**Figure 2:**
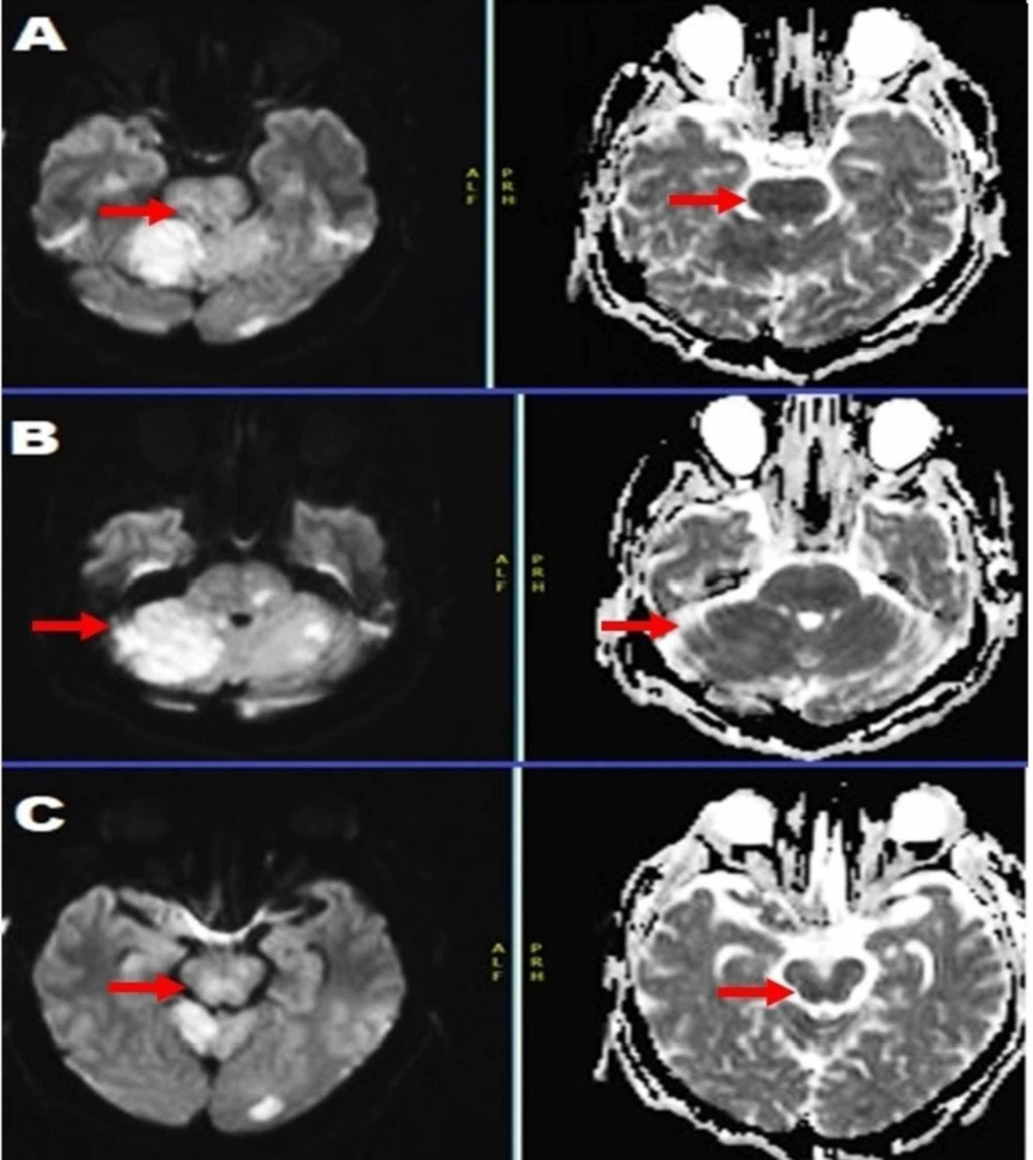
Multiple slices of MRI brain, diffusion-weighted images (DWI) 24 hours after successful mechanical thrombectomy, depicting evolving but less restriction in the pons on DWI with apparent diffusion coefficient (ADC) correlation.

MRI before discharge (Day 7 of the admission) showed a pronounced DWI restriction in the same areas involved in the hyperacute MRI (Figure 1) done at admission. However, now, the ADC hyper-intensity was less noticeable, with continued hazy and smoggy pontine signal changes on DWI (Figure 2).

Due to the patients’ claustrophobia, other detailed imaging cuts like fluid-attenuated inversion recovery (FLAIR) or T1 were not performed. He continued to recover with therapy clinically and had a notable improvement in his NIHSS of 3 for mild facial droop, mild aphasia, and wrong month on orientation, at the time of discharge (Day 8 of the admission). The patient had a modified Rankin Scale (mRS) score of 0 on admission and 1 at the time of discharge. On the two-month follow-up, he complained of right arm numbness in a small area and positional dizziness, with an NIHSS score of 0. The patient was offered a repeat MRI using research funds, however, he was not interested in pursuing further imaging. The comprehensive neurological exam did reveal subtle right arm motor and sensory changes but otherwise an intact neurological exam with stable non-ataxic gait. The workup for stroke etiology then was inconclusive, and the mRS score was 0.

## Discussion

This report focuses on the findings of hazy or smoggy DWI restriction or aa "smog sign" in the pons and the cerebellar peduncle regions in the emergent hyperacute MRI brain scan and its potential clinical implications. Most stroke centers offer intra-arterial thrombectomy (IAT) in patients with a posterior circulation stroke based on CT/CTA head findings rather than the hyperacute MRI brain findings as in this case. However, some advanced comprehensive stroke centers (CSC) also consider a hyper-acute MRI to assess for IAT candidacy in stroke cases presenting within the 12-hour window [10]. This case focuses on an important point: smoggy DWI changes (DWI restriction/ brightening) in the brainstem on a hyperacute MRI, correlating with a noticeable decrease in ADC (darkening). These early DWI/ADC MRI changes might indicate just partial damage to neurons manifesting as patchy areas of infarction in axons with regenerative potential. Hence, the BAO cases presented to CSC within 24 hours should still be considered for IAT in the hope of a good outcome, and the “smog sign” should not preclude the physicians from holding the best available treatment. The pontine lesions are known to be independently associated with early neurologic deterioration (END) [11]. The END, as indicated by DWI changes in MRI, is studied mainly for anterior circulation strokes and does correlate with clinical severity and outcome [12].

Basilar artery occlusion (BAO) is one of the most devastating forms of stroke and bears a grave prognosis if not successfully recanalized or revascularized [13-14]. Although in this case, successful IAT was performed within six hours of stroke onset, all major CSCs still perform IAT for life-threatening BAO cases as late as 24 hours from stroke onset [15]. Most of the decision-making for such stroke cases relies upon acute imaging findings, with DWI MRI brightening being considered an indication of cerebral ischemia, demonstrating ultrastructural changes secondary to brain death, differentiating them from edematous changes seen on T2-weighted images [16]. The reason for an aggressive approach to treatment with alteplase and IAT is the poor outcome in patients with a posterior circulation stroke without successful recanalization, with mortality reaching 80%-90% [17]. With an unknown time of stroke onset, imaging modalities play a crucial role in decision-making and on whether the patient is a suitable interventional candidate or not, as mentioned earlier.

Currently published studies are focused on anterior circulation LVOs, with insufficient data on posterior circulation stroke. Moreover, all these recent neuro-endovascular trials that benefited stroke patients had either Alberta stroke program early CT score (ASPECTS), multi-CT, or DWI/MRI as imaging modalities of selecting the pool of patients eligible for the IAT [7]. Van Houwelingen et al., in their study on the application of IAT in BAO, reported that 34 of 38 BAO cases (89%) who received IAT achieved an adequate recanalization with a favorable functional outcome in 19 (50%) cases and symptomatic ICH in only two (5%) cases [18]. These results are similar to the IAT group of the MR CLEAN trial and superior to the Basilar Artery International Cooperation Study (BASICS) registry cohort, suggesting the feasibility, safety, and efficacy of IAT in BAO but in contrast to the ENDOSTROKE study, collaterals do not seem to play a significant role. The ENDOSTROKE study showed that 79% of the BAO patients who were offered IAT achieved recanalization, with 34% having good and 42% moderate clinical outcome with 35% overall mortality [19]. This trial showed that along with age, hypertension, NIHSS, and the use of MRI before IAT collateral status predict the clinical outcome and recanalization in BAO. Bouslama M et al. suggested that the smoking status, low baseline NIHSS, and successful recanalization are associated with good clinical outcome in patients with posterior circulation stroke treated with IAT [20].

Patients with posterior circulation strokes and basilar occlusion or stenosis present with a broad spectrum of clinical findings, from double vision and slurred speech to comatose or locked-in state at the time of neurologic examination. Often, the timeline is variable, and it is always challenging when to go for IAT, as it is a very high-risk intervention. Our case, with the clinical exam suggestive of evolving locked-in syndrome at the time of presentation and imaging findings in the pons showing the "smog sign" or DWI restriction/brightening correlating with pronounced ADC signal changes in the same area complicated the treatment strategy. These findings might have misled the stroke neurologists and endovascular neuroradiologists caring for this patient against the provision of IAT, which was fortunately not the case. The prompt provision of IAT and successful recanalization led to the full recovery of the subject. This case demonstrates that there could be an early DWI restriction in areas where axonal tracts predominate, which may appear like a "smog sign" on DWI with rather more pronounced amplification of ADC, misleading the healthcare teams in jumping to the early conclusion of an irreversible brain injury, which in reality might be reversible. Hence, for the BAO cases who arrive early to the CSC, even with as severe as the locked-in syndrome with having a “smog sign” on DWI MRI on presentation should still be offered the IAT in hopes of a favorable outcome. As evidenced by this case, emergency physicians, stroke neurologists, radiologists, and neuro-endovascular experts need to be aware of the “smog” sign in the pons or other tract areas (e.g., corona radiata) to allow prompt treatment by IAT to the eligible patients. Further, large-scale studies are needed to validate these findings in case series and prospective studies for the application of IAT in patients with BAO presenting with variable clinical features and despite the presence of a “smog sign.”

## Conclusions

We propose that the DWI-MRI changes in acute ischemic stroke behave differently in tract areas with a density of nerve axons, manifesting as a hazy or smoggy appearance: the “smog sign” on DWI. In hyper-acute MRI, "hazy" or "smoggy" diffusion restriction on DWI in different axonal tract areas like the pons can correlate with good functional outcomes if successful reperfusion therapy is offered as early as possible.
